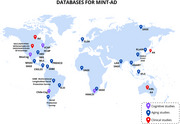# Multimodal Interpretable Transformer for AD

**DOI:** 10.1002/alz70861_108625

**Published:** 2025-12-23

**Authors:** Paola Ruiz Puentes, Néstor F. González García, Daniel Crovo, Felipe Sánchez Mejía, María C. Ibáñez, Pablo Arbeláez, Ram Mukunda

**Affiliations:** ^1^ IGC Pharma, Bogotá, Bogotá D.C Colombia; ^2^ Syndeo International S.A.S, Bogotá, Bogotá D.C Colombia; ^3^ IGC Pharma, Potomac, MD USA

## Abstract

**Background:**

Artificial intelligence (AI) has significantly improved diagnostic accuracy in fields such as cancer, diabetes, and cardiopulmonary disease. It holds similar promise for Alzheimer’s disease (AD), where underdiagnosis remains a major challenge (false negatives = 11.9%). Addressing this requires models trained on diverse datasets that capture global variability in socioeconomic and health conditions. We present a methodology to develop the Multimodal Interpretable Transformer for AD (MINT‐AD) to support early risk detection and personalized intervention.

**Methods:**

MINT‐AD is trained on a corpus of 32 databases from North America, South America, Europe, Africa, Asia, and Oceania. This includes 14 longitudinal studies following the Health and Retirement Study (HRS) protocol with basic cognitive measures and 4 cognitive‐ancillary cohorts; 6 further aging cohorts—including 3 multicountry studies; 1 separate cognitive study; 10 clinical Alzheimer’s and dementia registries; and a comprehensive genetic and epigenetic Alzheimer’s dataset. MINT‐AD’s architecture combines a Mixture‐of‐Experts framework with LLMs trained via Chain‐of‐Thought on patient narratives for intuitive interaction, alongside time‐series sequence models for personalized early Alzheimer’s risk prediction. The model integrates clinical, genetic, socioeconomic, and lifestyle data to identify interpretable risk clusters to simulate long‐term intervention impacts of modifiable interventions.

**Results:**

Seven HRS‐related databases have been harmonized by the Global2Aging initiative, with seven more in progress by our team. We have harmonized five clinical databases with ongoing efforts for the remainder. Using state‐of‐the‐art (SOTA) transformer architectures, we have developed two early modules: one LLM‐based model predicts MMSE from sociodemographic data, and the other predicts dementia diagnosis using clinical and imaging data.

**Conclusion:**

By leveraging these diverse datasets and advanced AI models, MINT‐AD aims to become a foundation model for Alzheimer’s prediction and classification. Designed as a clinical support tool, it will enhance early detection, risk stratification, and intervention planning without replacing standard neuropsychological assessments. At the population level MINT‐AD will enable risk mapping across largescale epidemiological variables like education, healthcare access, and environmental exposures, informing public health strategies for targeted AD prevention.